# Outpatient Parenteral Antimicrobial Therapy in a Setting with High Prevalence of Multidrug Resistance: Challenges, Opportunities, and Stewardship Considerations

**DOI:** 10.3390/antibiotics15060594

**Published:** 2026-06-10

**Authors:** Giacomo Ciusa, Giuseppe Pipitone, Bianca Catania, Giulia Coniglione, Marilee Goad, Claudia Imburgia, Preziosa Scordo, Antonio Albanese, Antonio Cascio, Chiara Iaria

**Affiliations:** 1 Infectious Diseases Unit, ARNAS Civico-Di Cristina Hospital, 90127 Palermo, Italy; giac.ciusa@gmail.com (G.C.); marileegoad@gmail.com (M.G.); claudia.imburgia@arnascivico.it (C.I.); chiara.iaria@arnascivico.it (C.I.); 2 Foreign Family Health Center, Department of Primary Care, AUSL-IRCCS of Reggio Emilia, 42100 Reggio Emilia, Italy; 3 Department of Health Promotion, Mother and Child Care, Internal Medicine and Medical Specialties, G D’Alessandro, AOUP P. Giaccone, University of Palermo, 90127 Palermo, Italy; antonio.cascio03@unipa.it; 4 Infectious Diseases Unit, Papardo Hospital, 98120 Messina, Italypreziosascordo@aopapardo.it (P.S.); antonioalbanese@icloud.com (A.A.)

**Keywords:** OPAT, antimicrobial stewardship, multidrug resistance, outpatient antimicrobial therapy, MDR, Southern Italy

## Abstract

**Background**: Outpatient parenteral antimicrobial therapy (OPAT) is increasingly used for complex infections, but data from settings with high prevalence of multidrug-resistant organisms (MDROs) are limited. **Methods**: We conducted a retrospective observational study of patients treated with OPAT in two tertiary-care hospitals in Southern Italy between 2024 and 2025. **Results**: Among 119 patients, 51.2% had MDRO infections. Clinical recovery was achieved in 98/109 (89.9%) patients, with low rates of relapse (11/109, 10.1%), hospital readmission (19/109, 17.4%), and adverse events. Recovery, relapse, adverse events, and hospital readmission rates appeared similar between MDRO and non-MDRO groups, and no statistically significant differences were found. **Conclusions**: These findings suggest that OPAT may be safely and effectively delivered in high-MDRO-prevalence settings when provided within structured, specialist-led hospitals, but it should be interpreted with caution due to the limited sample size and reduced statistical power. The study primarily contributes a detailed characterization of feasibility and stewardship challenges rather than a definitive efficacy comparison, which requires future prospective studies.

## 1. Introduction

Outpatient Parenteral Antimicrobial Therapy (OPAT) represents an alternative to hospital admission for treating complicated infections in clinically stable patients. It has been demonstrated its safety, efficacy [1], cost-effectiveness [2], and good tolerability [3], although this evidence came from settings with low prevalence of multidrug-resistant organisms (MDROs).

Moreover, the outpatient setting warrants specific attention to safety, therapeutic adherence, and antimicrobial stewardship [4,5,6].

In Italy, OPAT delivery is not standardized at a national level. Services are organized on a hospital-by-hospital basis, typically under the supervision of infectious diseases specialists, and patients are managed through outpatient clinic visits rather than nurse home visits. This organizational model affects the frequency and intensity of clinical monitoring, the feasibility of continuous infusion regimens, and the resources available to support patients with complex drug administration schedules.

Southern Europe, particularly Italy and Sicily, faces a disproportionate burden of resistant Gram-negative bacteria, including carbapenem-resistant producers and extended-spectrum beta-lactamase (ESBL)-producing organisms, creating a challenge for OPAT and drug selection.

These epidemiological characteristics create specific challenges for OPAT: the agents required to treat MDR pathogens are frequently restricted to intravenous formulations, require continuous infusion schedules for optimal pharmacodynamic target attainment, and carry a higher cost and toxicity burden than those used in standard OPAT programmes.

Several reasons might lead to worse OPAT outcomes in MDRO infections compared to susceptible infections: (i) limited antibiotic options compatible with outpatient delivery; (ii) greater reliance on invasive vascular access (midline catheters) for continuous infusion; (iii) the need for novel or reserve agents with less well-characterized outpatient safety profiles; and (iv) the higher clinical complexity of patients who acquire MDR infections. Whether these concerns translate into measurable outcome differences in a real-world OPAT setting is unknown.

The aim of this study was to investigate the challenges and opportunities of OPAT in a high-MDRO-prevalence setting by analysing a cohort of patients treated in two tertiary-care hospitals in Sicily. Specifically, we sought to: (1) characterize the safety and efficacy of OPAT in patients with microbiologically documented MDRO infections; (2) assess whether antimicrobial resistance profile impacts clinical outcomes; and (3) evaluate the feasibility of structured OPAT delivery in resource-limited, high-resistance epidemiological contexts. Given the small sample size, the study is primarily intended as a hypothesis-generating description rather than a definitive comparative analysis.

## 2. Materials and Methods

### 2.1. Study Design and Setting

This retrospective observational cohort study included patients who used OPAT services at the Infectious Diseases Unit clinics of two tertiary-care hospitals (ARNAS Civico–Di Cristina–Benfratelli of Palermo and AO Papardo of Messina) between 1 April 2024, and 31 July 2025. Patients were enrolled both upon discharge after hospital admission and as direct outpatients.

### 2.2. OPAT Programme Organization

In both participating centres, OPAT was delivered through dedicated outpatient infectious diseases clinics. All patients attended scheduled visits for antibiotic administration and clinical review by an infectious diseases physician and supervised nurse during the whole week. Wound care and dressing changes were provided by the nursing team where clinically required. OPAT was defined as parenteral antimicrobial therapy delivered entirely in the setting.

### 2.3. Patient Population and Eligibility Criteria

Eligibility criteria included age ≥ 18 years, requirement for parenteral antimicrobial therapy as determined by an infectious diseases specialist, clinical stability (defined as absence of septic shock or haemodynamic instability requiring vasopressor support), and ability to attend scheduled follow-up visits.

### 2.4. Antibiotic Selection and Vascular Access

Antibiotic treatments were administered according to institutional protocol. The choice between continuous infusion and intermittent once-daily dosing was guided by: (i) the pharmacokinetic/pharmacodynamic (PK/PD) target of the antibiotic (time-dependent agents preferred for continuous infusion; concentration-dependent agents for once-daily regimens); (ii) antibiotics’ physicochemical stability (agents stable for ≥24 h in elastomeric devices were candidates for continuous infusion); and (iii) choice of the patient. Peripheral venous catheters were used for once-daily intermittent regimens, typically inserted at each visit or maintained for no more than 3 days. Midline venous catheters were placed for patients requiring continuous elastomeric infusion and were managed according to institutional protocol, including regular flushing with heparinised saline, daily site inspection at each outpatient visit, and replacement in the event of occlusion, phlebitis, or suspected infection.

### 2.5. Data Collection

For each enrolled patient, demographic characteristics (age and sex), comorbidities, and the Charlson Comorbidity Index (CCI) were collected. Clinical data included baseline body temperature (°C), white blood cell count (WBC), and C-reactive protein (CRP, mg/dL) at treatment initiation. Microbiological isolates and specimen types were recorded. Antibiotic prescriptions were recorded, including drug, dosage, vascular access type, and treatment duration. Adverse events occurring during therapy were systematically registered at each outpatient visit by the treating physician and categorized as either device-related (catheter-related) or drug-related. A formal validated severity grading scale was not applied; this represents a methodological limitation.

### 2.6. Outcome Definitions

Clinical outcomes were defined as: (i) recovery (completion of therapy with complete regression of signs and symptoms as assessed by the infectious diseases specialist at the end-of-therapy outpatient evaluation); (ii) symptom persistence or clinical relapse within 90 days; (iii) hospital admission or readmission for any cause within 90 days; and (iv) death. Follow-up was performed through scheduled outpatient clinic visits at 90 days after completion of therapy.

### 2.7. Patient Stratification and Statistical Analysis

Patients were stratified into two groups: those infected with MDROs [including ESBL-producing, KPC-, and VIM-producing Gram-negative bacteria, multidrug-resistant Pseudomonas aeruginosa, and MRSA] and those infected with fully susceptible or mono-resistant pathogens. Patients without a documented microbiological isolate (classified as ND, n = 27) were included in the overall descriptive analysis but excluded from the MDRO/non-MDRO subgroup comparison, as their resistance status could not be ascertained. Fisher’s exact test or chi-square test was used for categorical variables; the Mann–Whitney U test was used for continuous variables.

## 3. Results

### 3.1. Patient Enrolment and Demographics

During the study period, 139 patients accessed OPAT services. Of these, 119 were included in the final analysis; 20 were excluded due to incomplete data or loss to follow-up. Of the 119 included patients, 90-day follow-up outcome data were available for 109 (91.6%); the remaining 10 did not attend the scheduled 90-day outpatient follow-up visit and were excluded from outcome analyses.

Among included patients, 67/119 (56.3%) were male, with a median age of 67 years (IQR 51–76). The median CCI was 3 (IQR 2–5.5). The most frequent comorbidities were ischaemic heart disease (39/119, 32.8%), diabetes mellitus (35/119, 29.4%), chronic pulmonary disease (26/119, 21.8%), solid tumours (23/119, 19.3%), chronic heart failure (21/119, 17.6%), peripheral vascular disease (19/119, 16.0%), and chronic kidney disease (8/119, 6.7%).

### 3.2. Infectious Diagnoses and Microbiology

The most common infectious diagnoses were cUTI (35/119, 29.4%), osteomyelitis (30/119, 25.2%), pneumonia (20/119, 16.8%), ABSSSI (17/119, 14.3%), and endocarditis (7/119, 5.9%). At baseline, 25% of patients had a body temperature > 38 °C. The median WBC was 7600 cells/µL (IQR 6300–9000) and the median CRP was 1.22 mg/dL (IQR 0.5–2.43).

A microbiological isolate was obtained in 92/119 (77.3%) patients. Among the 27 (22.7%) patients without microbiological documentation, empirical antibiotic therapy was prescribed by an infectious diseases specialist on the basis of clinical syndrome, local epidemiological patterns, and prior microbiological history where available. These patients were included in the overall descriptive analysis but excluded from the MDRO/non-MDRO subgroup comparison, given the impossibility of ascertaining their resistance status. The most frequently isolated pathogens were *P. aeruginosa* (27/92, 29.3%), *K. pneumoniae* (23/92, 25.0%), and *S. aureus* (15/92, 16.3%). Polymicrobial infections accounted for 12/92 (13.0%) of isolates. Overall, 61/119 (51.2%) patients were classified as having MDR infections (Table 1).

### 3.3. Antibiotic Therapy

Of the 119 patients, 32 (26.9%) received continuous antibiotic infusion via elastomeric pumps, 14 (11.8%) received long-acting lipoglycopeptides, and the remaining 73 (61.3%) were managed with intermittent intravenous regimens administered through peripheral venous catheters. Continuous infusion was used most commonly for ceftolozane/tazobactam or piperacillin/tazobactam, primarily targeting MDR Gram-negative pathogens in osteomyelitis, ABSSSI, cUTI, and pneumonia. Long-acting lipoglycopeptides (dalbavancin or oritavancin) were prescribed in 8 patients for ABSSSI and in 6 patients off-label for osteomyelitis. Once-daily and intermittent regimens included aminoglycosides, glycopeptides, extended-spectrum cephalosporins, and other beta-lactams, used principally for susceptible or non-MDR infections. The median treatment duration was 14 days (IQR 7–14).

### 3.4. Adverse Events

Drug-related adverse reactions occurred in 5/114 (4.4%) patients, including 4 cases of nausea and one case of Clostridioides difficile colitis. No allergic reactions were observed, and no drug-related adverse events required hospital admission. Catheter-related complications occurred in 12/118 (10.2%) patients, including one midline-associated venous thrombosis requiring anticoagulation. No deaths occurred.

### 3.5. Clinical Outcomes

At the end of therapy, 98/109 (89.9%) patients achieved clinical recovery, while 11/109 (10.1%) experienced symptom persistence or relapse. Overall, 19/109 (17.4%) required hospital admission or readmission within 90 days.

### 3.6. Comparison Between MDRO and Non-MDRO Groups

Among the 109 patients with available 90-day outcome data and microbiological documentation (Table 2), 25 (22.9%) had susceptible (non-MDRO) infections and 58 (53.2%) had MDR infections (the remaining 26 had no microbiological isolate and were excluded). The MDRO and non-MDRO groups did not differ significantly in age (median 68 vs. 69 years; *p* = 0.728) or sex distribution (52.5% vs. 46.2% male; *p* = 0.644). The MDR group showed a slightly higher median CCI (4, IQR 2–6 vs. 3, IQR 2–4; *p* = 0.142).

Once-daily regimens were more frequently used for susceptible infections, whereas continuous-infusion beta-lactams and midline catheters were more common in MDR cases (*p* = 0.011).

## 4. Discussion

Italy’s epidemiology is characterized by a high prevalence of MDR organisms [7], as reflected by our cohort, in which MDR infections accounted for over half of all cases. In our cohort, the patients with MDR infections were similar to those with susceptible infections in terms of age and sex but had a slightly higher comorbidity burden, supporting the association between clinical complexity and MDR infections even in high-prevalence settings [8,9]. The principal contribution of this study is characterization of the feasibility, safety profile, and stewardship challenges of OPAT in a MDR epidemiological context.

Most published OPAT data originate from Northern Europe and North America, where MDR epidemiology differs substantially from Southern Italy, one of the European regions with the highest rates of carbapenem-resistant Gram-negative bacteria [7]. Our study therefore addresses a distinct and underrepresented clinical context, where OPAT programmes must accommodate a disproportionate burden of difficult-to-treat pathogens requiring novel or broad-spectrum agents.

OPAT was confirmed to be safe and effective in this cohort, including among patients with MDR infections. Extending OPAT to this population required the use of broad-spectrum and high-cost antibiotics and greater reliance on midline catheters and elastomeric infusion devices. Long-acting lipoglycopeptides allowed inclusion of patients who would otherwise be excluded due to logistical barriers, while protected beta-lactams were chosen for select Gram-negative infections based on outpatient pharmacological stability [10,11,12].

Although recovery, relapse, adverse events, and hospital readmission rates appeared comparable between the MDRO and non-MDRO groups, with no statistically significant differences detected; these findings should be interpreted with caution due to the limited sample size—particularly in the non-MDRO subgroup (n = 25)—and the consequent reduced statistical power and risk of Type II error (Table 2).

In our study, cUTI was more frequent among MDR cases. Its overrepresentation in the MDR subgroup may have partially attenuated differences in outcome between groups, as cUTI may have a lower risk of treatment failure.

From a stewardship perspective, this study highlights usefulness of OPAT in high-MDRO settings: specialist-led prescription, clinical examination of the patients at each outpatient visit, and the avoidance of hospitalization—a recognized risk factor for healthcare-associated and MDR infections [13,14,15]—represent benefits in terms of stewardship. Quantifying the extent of this practice requires individual-level prescribing data unavailable in the present dataset; we acknowledge this as a priority for future OPAT registries. OPAT programmes in high-MDRO settings should integrate systematic de-escalation review at each outpatient visit and microbiological reassessment when clinically indicated.

The economic implications of OPAT in this context are relevant. Published UK cost-effectiveness data demonstrate savings of approximately £1000–5000 per OPAT episode compared to inpatient care for six key diagnoses [2]. Pharmacoeconomic modelling in high-MDRO Italian OPAT settings is lacking and represents an important research gap.

Nevertheless, the ecological impact of extending broad-spectrum and novel antibiotic use to the outpatient setting warrants attention. In high-MDRO-prevalence contexts, the risk of further resistance selection through prolonged community exposure to last-resort agents is real, and OPAT programmes should integrate systematic stewardship oversight, including de-escalation review at each outpatient visit and microbiological reassessment when clinically indicated.

By avoiding hospital-related infectious and non-infectious complications, OPAT may preserve patient-centered outcomes such as functional status and cognitive performance [16]. Additionally, economic savings associated with reduced hospitalization may justify the use of costly antimicrobial agents [2]. OPAT and long acting glycolipopeptides use should therefore be encouraged, even in the context of high prevalence of MDRO, to shift the management of complex infections toward outpatients setting [17,18].

### Limitations

Study limitations include the retrospective design, potential selection and allocation bias, absence of a control group, and the combined analysis of Gram-positive and Gram-negative MDR infections despite their differing risk profiles. The pooling of biologically and therapeutically heterogeneous MDRO subgroups (CPO, ESBL-producers, MDR *P. aeruginosa*, MRSA) is an important constraint on the interpretability of comparative findings. The relatively small sample size, particularly in the non-MDRO subgroup (n = 25), limits statistical power and raises the risk of Type II error; the absence of statistically significant differences should not be interpreted as evidence of clinical equivalence. Adverse events were not formally graded using a validated severity scale (e.g., CTCAE) due to the retrospective nature of the study. Oral step-down therapy was not systematically tracked, and data on antibiotic use and resistance outcomes in patients who failed therapy could not be retrieved, precluding analysis of treatment failure predictors. The exclusion of 32 patients without microbiological documentation from the subgroup comparison may introduce selection bias. Additionally, 10 patients (8.4%) did not complete the 90-day follow-up and were excluded from outcome analyses, potentially underestimating adverse outcomes.

## 5. Conclusions

Our data reinforce the feasibility of OPAT in a high-MDRO-prevalence setting, with low rates of adverse events and hospital readmission. Although recovery rates appeared comparable between MDRO and non-MDRO infections, the limited sample size precludes definitive conclusions about comparative efficacy. The organism resistance profile alone should not preclude OPAT consideration, provided that specialist antimicrobial oversight is maintained, and regimens are optimized for organism and host factors. OPAT should be integrated into antimicrobial stewardship strategies to optimize treatment of complicated infections while limiting hospital exposure and reducing secondary resistance development. Future prospective studies and cost-effectiveness analyses in high-MDRO-prevalence settings, with standardized severity grading, systematic microbiological follow-up, and subgroup-level outcome reporting, are warranted.

## Figures and Tables

**Table 1:** Infectious diseases diagnosed, types of specimens analyzed, and microbiological isolates are listed. cUTI: complicated urinary tract infection; ABSSSI: Acute Bacterial Skin and Skin Structure Infections; BAL: bronchoalveolar lavage.

Infectious Diseases	Microbiological Specimen	Microorganism
cUTI	Urine culture 33/35 (94.3%)	*K. pneumoniae* 19/35 (54.3%); *E. coli* 10/35 (28.6%); *P. aeruginosa* 3/35 (8.6%); *P. mirabilis* 2/35 (5.7%); *E. faecalis* 2/35 (5.7%); *E. cloacae* 1/35 (2.8%).
Osteomyelitis	Bone biopsy 20/29 (69%); Swab culture 5/29 (17.2%); Blood culture 1/29 (3.4%)	*P. aeruginosa* 12/29 (41.4%); *S. aureus* 6/29 (20.7%); *S. epidermidis* 4/29 (13.8%); *E. faecalis* 2/29 (6.9%); Others 7/29 (24.1%).
Pneumonia	Sputum culture 7/20 (35%); BAL culture 4/20 (20%); Blood culture 2/20 (10%)	*P. aeruginosa* 7/20 (35%); *Aspergillus* spp. 3/20 (15%); *S. pneumoniae* 2/20 (10%); *K. pneumoniae* 1/20 (5%);
ABSSSI	Swab culture 8/17 (47%); Biopsy culture 4/17 (23.5%)	*S. aureus* 6/17 (35.3%); *P. aeruginosa* 5/17 (29.4%) *P. mirabilis* 2/17 (11.8%); *K. pneumoniae* 1/17 (5.9%)
Endocarditis	Blood culture 2/7 (28.6%); Valve culture 1/7 (14.3%); intracardiac device 1/7 (14.3%)	*Alpha-haemolytic Streptococci* 2/7 (28.6%); *S. warneri* 1/7 (14.3%); *K. pneumoniae* 1/7 (14.3%)
Septic arthritis	articular fluid culture 1/2 (50%)	*S. aureus* 1/2 (50%)
Prostatitis	Sperm culture 1 (100%)	*P. aeruginosa* and *K. pneumoniae* 1 (100%)

**Table 2:** Clinical outcomes, type of antibiotic administration (single daily dose versus continuous infusion with elastomeric pumps), and adverse reactions related to the drug and the catheter are listed. Differences between the two patients’ cohorts, the group of those infected by non-multi-drug-resistant organisms, and the group of those infected by multidrug-resistant organisms are shown. MDRO: multi-drug resistant organism.

Clinical Outcomes	Overall	Non-MDRO	MDRO	*p*-Value
Clinical Recovery	98/109 (89.9%)	23/25 (92.0%)	50/58 (86.2%)	0.716
Persistence/Relapse within 90 days	11/109 (10%)	2/25 (8.0%)	8/58 (13.8%)	0.716
Hospital readmission within 90 days	19/109 (17.4%)	5/23 (21.7%)	9/58 (15.5%)	0.525
Antibiotic treatment				
One daily dose antibiotic	88/119 (73.9%)	19/26 (73%)	33/61 (54.1%)	0.151
Antibiotic in continuous infusion	32/119 (26.9%)	3/26 (11.5%)	25/61 (41%)	0.011
Long-acting glycolipopeptides	14/119 (11.8%)	4/26 (15.4%)	3/61 (4.9%)	0.190
Adverse Events				
Drug-related adverse reaction	5/114 (4.4%)	0/26 (0%)	2/58 (3.4%)	1.0
Catheter-related adverse reaction	12/118 (10.2%)	2/26 (7.7%)	7/61 (11.5%)	0.719

## Data Availability

The data presented in this study are available on request from the corresponding authors.
